# Case report: Unilateral panuveitis as a manifestation of Alport syndrome in a Chinese pediatric patient

**DOI:** 10.3389/fgene.2022.934829

**Published:** 2022-11-14

**Authors:** Yu Tian, Xiaochuan Wu, Yongzhen Li, Wenbin He, Zibin Liu, Frank L. Myers, Liang Zhou

**Affiliations:** ^1^ Department of Ophthalmology, The Second Xiangya Hospital, Central South University, Changsha, Hunan, China; ^2^ Department of Ophthalmology, Hunan Children’s Hospital, Changsha, Hunan, China; ^3^ Department of Pediatrics, The Second Xiangya Hospital, Central South University, Changsha, Hunan, China; ^4^ Institute of Reproductive and Stem Cell Engineering, School of Basic Medical Science, Central South University, Changsha, Hunan, China; ^5^ Department of Ophthalmology and Visual Sciences, University of Wisconsin-Madison School of Medicine and Public Health, Madison, WI, United States

**Keywords:** Alport syndrome, uveitis, *COL4A5*, pediatric, collagen

## Abstract

**Purpose:** The study aimed to report a rare case of a patient with Alport syndrome, which was manifested as unilateral non-infectious uveitis after bilateral cataract surgery.

**Methods:** A case report.

**Results:** A 2-year-old boy was diagnosed with unilateral panuveitis based on the clinical and multimodal imaging findings. Intraocular fluid samples for metagenomic next-generation sequencing (mNGS) and microbial culture were negative. However, urine tests found proteinuria and microscopic hematuria. Pathologic findings of the kidney revealed a thickened membrane, and a diagnosis of Alport syndrome was considered. Gene analysis found deletions in exon 1 of *COL4A5* and exons 1 and 2 of *COL4A6*. The uveitis resolved gradually, following the administration of oral steroids.

**Conclusion:** Uveitis may be an ocular manifestation of Alport syndrome.

## Introduction

Alport syndrome (AS) is a hereditary disease that involves the basement membrane of the glomerulus, cochlea, lens capsule, and retina. The ocular manifestations reported include anterior lenticonus, “dot and fleck” retinopathy, temporal macular thinning, and vitelliform maculopathy (Savige and ColvilleOpinion, 2009). In this report, we present a case of Alport syndrome manifested as unilateral non-infectious uveitis, which was successfully treated with steroids.

## Case report

A 2-year-old boy with redness and hazy vitreous in the right eye was referred to our clinic. The patient underwent bilateral cataract surgeries 1 year ago. A review of the surgical recording and ultrasound revealed previous total cataracts, as well as whitening of nuclei in the cortex of both eyes. One month ago, in the cataract clinic, his mother reported redness and blurred vision in his right eye, and endophthalmitis was suspected.

**FIGURE 1:**
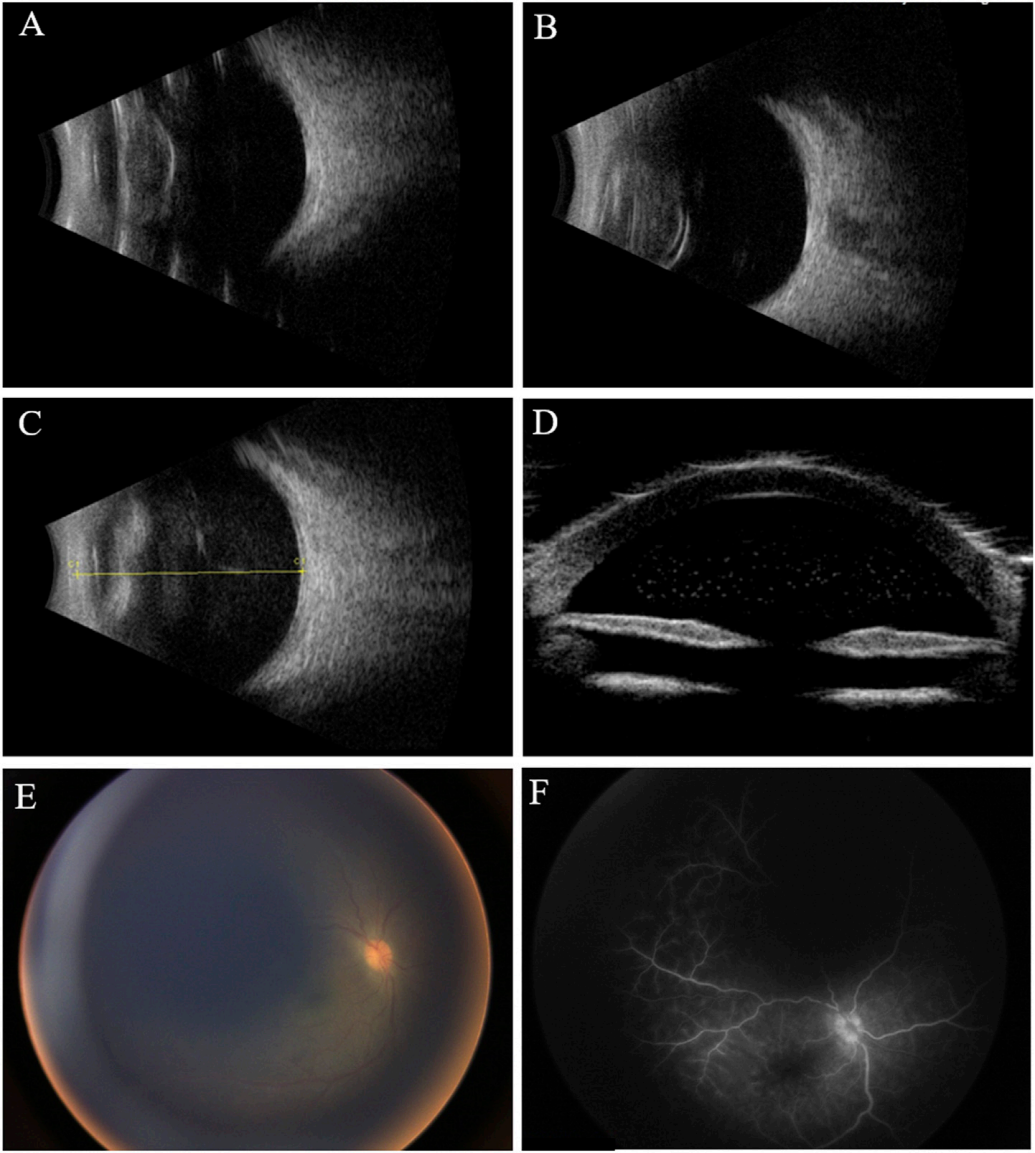
Clinical examination of the eyes at different times. **(A,B)** B-scan ultrasound shows opacified but otherwise normal-shaped lenses in both eyes before cataract surgeries. **(C)** B-scan ultrasound shows opacified vitreous 1 year after the cataract surgery; **(D)** ultrasound biomicroscopy (UBM) shows hyperechogenic dots in the anterior chamber; **(E)** fundus photography reveals hazy vitreous and congested optic nerve head; **(F)** venous phase of fluorescein angiography shows leakage of retinal capillaries and a hyperfluorescent optic nerve head.

Ophthalmic examination under anesthesia revealed normal intraocular pressure in both eyes (11 mmHg OD and 12 mmHg OS). The right eye had combined ciliary and conjunctival congestion, generally clear cornea, absent lens, and opacified vitreous. Ultrasonography demonstrated multiple hyperechogenic dots in the anterior chamber and vitreous opacity, indicating inflammation (Figures 1A,B). Wild-field fundus photography revealed a congested optic nerve head through the opacified vitreous (Figure 1C). Fluorescein angiography showed the retinal capillaries and hyperfluorescent optic disc leakage at the venous phase (Figure 1D). A single dose of combined vancomycin (1 mg/0.1 ml), ceftazidime (2.2 mg/0.1 ml), and dexamethasone (0.5 mg/0.1 ml) was injected consequently into the vitreous cavity. The aqueous fluid was drawn and analyzed *via* metagenomic next-generation sequencing (mNGS). Based on a published protocol, this technology can detect 20,343 different types of microbes including bacteria, viruses, fungi, parasites, and other pathogens (Beijing Zhide Medical Laboratory Co., Ltd., an Illumina NextSeq platform) (Qian et al., 2022). Briefly, the intraocular fluid sample was extracted and purified using a TIANamp micro DNA kit (TIANGEN, Beijing, P.R.C). Qualified DNA was applied to construct DNA libraries using the QIASeq™ Ultralow Input Library Kit (QIAGEN, Hilden, Germany). Qualified libraries were sequenced on a NextSeq 550 platform (Illumina, San Diego, United States). After obtaining the sequencing data, high-quality data were generated after filtering out the adapter, low-quality, low-complexity, and shorter reads. Next, human reads were removed by mapping the reads to the human reference genome by SNAP software. The remaining data were aligned to the microbial genome database using Burrows–Wheeler alignment. However, the result was negative. Inflammatory factor analysis of the aqueous fluid found elevated levels of VEGF at 97.7 pg/ml (normal range 1–40), basic fibroblast growth factor (BFGF) of 4.9 pg/ml (normal range <1.0), interleukin (IL)-6 of 7524.5 pg/ml (normal range 1.0–50.0), vascular adhesion molecule (VCAM) of 11066.9 pg/ml (normal range 200–1,000), and IL-8 of 3023.1 pg/ml (normal range 0–20.0). Finding no obvious cause for the vitreous inflammation, a vitreous sample was collected *via* vitrectomy. The sample was cultured for bacteria and fungi, and T-SPOT and procalcitonin tests were conducted to rule out tuberculosis and sepsis. All the results came out negative.

**FIGURE 2:**
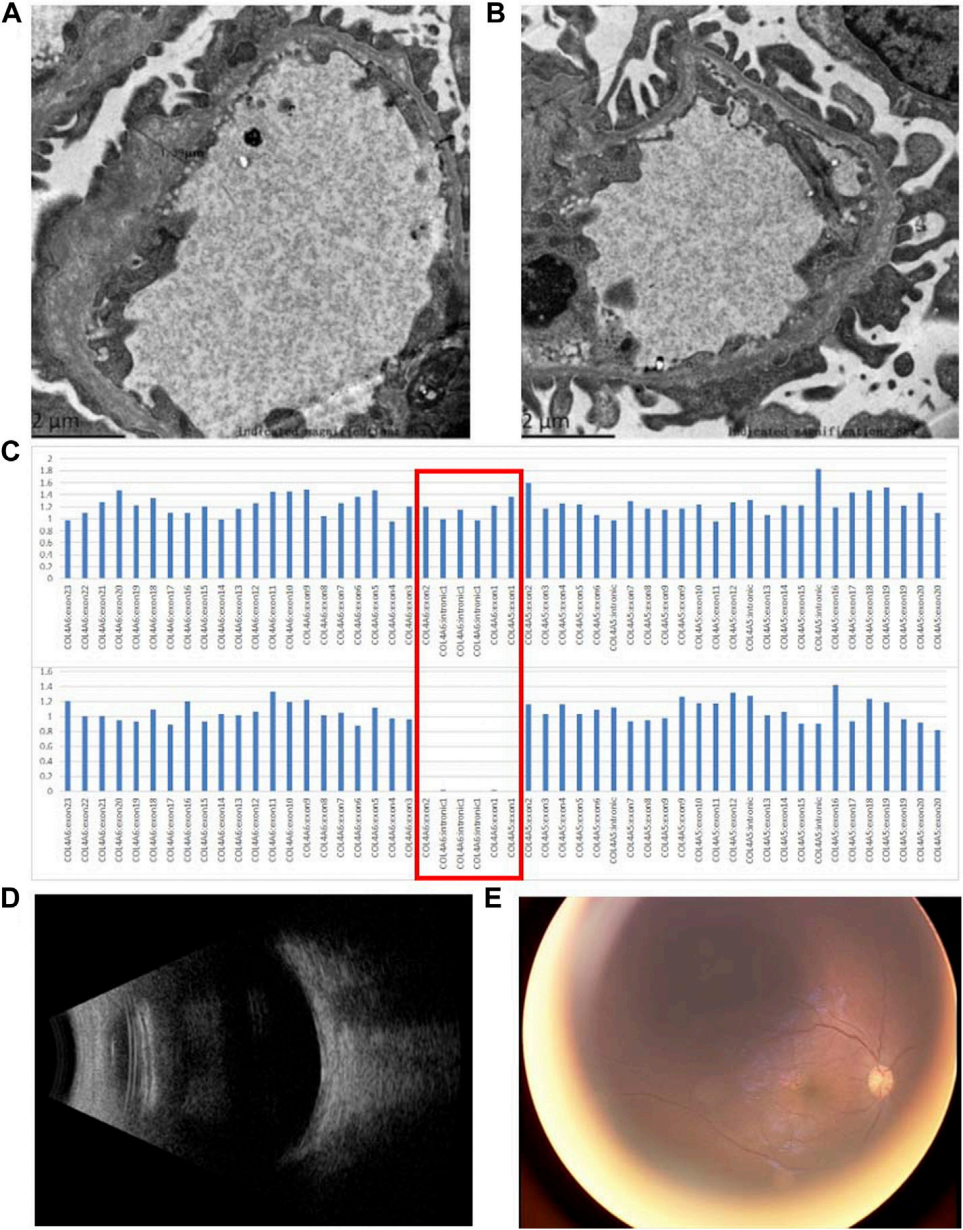
Pathological and genetic findings of the patient and the ocular findings after the treatment with steroids. **(A,B)** Electron microscopic analysis of a renal biopsy shows mesangial proliferation and thickening of the glomerular basement membrane (asterisks); **(C)** WES revealed that a deletion encompassing exon 1 of *COL4A5* and exons 1 and 2 of *COL4A6* was found in the patient. The red box indicates exon 1 of *COL4A5*, intron 1, and exons 1 and 2 of *COL4A6*. The top panel and bottom panels show the copy number of *COL4A5* and *COL4A6* from a normal individual and the patient, respectively; **(D)** B-scan ultrasound reveals a clear vitreous 1 month after treatment with steroids; **(E)** fundus photography shows resolved vitreous opacity and a clear optic nerve head.

**FIGURE 3:**
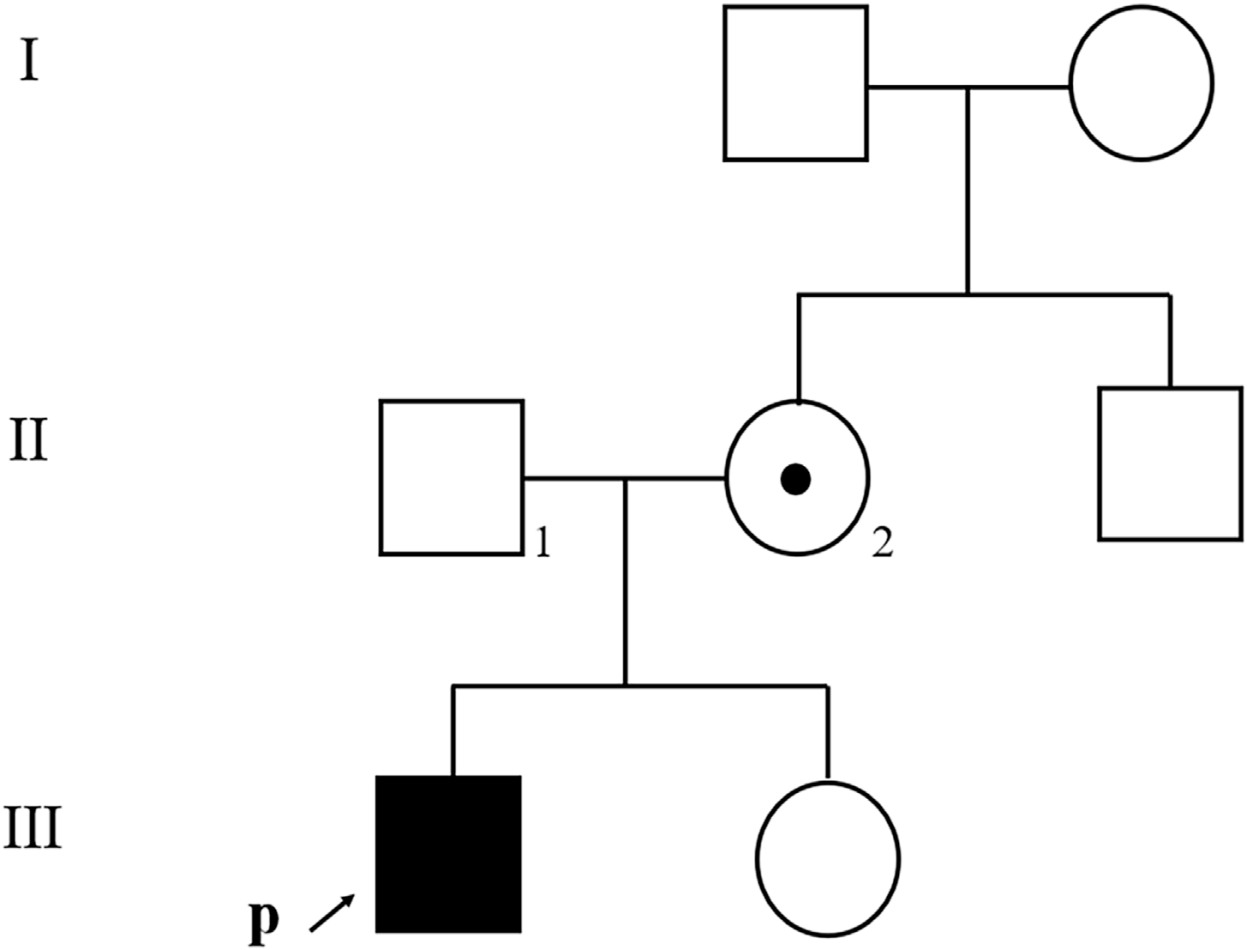
Family pedigree chart of the patient. An affected family member is indicated with filled symbols; unaffected relatives are indicated by open symbols; a heterozygous carrier is indicated with a dot in the middle of the symbol. Arrows indicate the proband. Numbers are allotted to the family members whose DNA samples were used in this study.

The patient was generally well-developed with no obvious abnormality on systemic examination. The family history was not remarkable. A systemic evaluation was performed when a diagnosis of unilateral non-infectious uveitis was considered. The immunological and rheumatological evaluation was not contributory. However, urinalysis revealed proteinuria and microscopic hematuria. The patient was then transferred to the pediatric department for further diagnosis. Electron microscopic analysis of a renal biopsy showed mesangial proliferation and thickening of the glomerular basement membrane (Figures 2A,B). Based on the pathology, Alport disease was suspected. The patient was subjected to whole-exome sequencing (WES) using the HiSeq X Ten sequencing platform (Illumina, San Diego, California, United States) and by MyGenostics, as described previously (Yang et al., 2022). A deletion in the X chromosome (Xq22.3: NC_000023.11:g.(107681122_107683515)del) encompassing exon 1 of *COL4A5* and exons 1 and 2 of *COL4A6* (Figure 2C) was found, which was confirmed by multiplex ligation-dependent probe amplification (MLPA). No hearing abnormalities were detected by audiometry. The patient exhibited no signs of dysphagia; however, close follow-ups are advised for this young patient. The child’s mother had neither microscopic hematuria nor ocular manifestation. A family pedigree chart of the patient is shown in Figure 3.

Oral prednisone was prescribed for uveitis, starting at 15 mg/day and was gradually tampered. One month after steroid treatment, inflammation in the vitreous cavity resolved. Congestion of the optic nerve head was resolved, as shown in fundus photography (Figure 2D). However, proteinuria and hematuria still existed.

## Discussion

Alport syndrome was first reported in a family with both nephritis and deafness by Arthur C. Alport in 1927 (Alport, 1927). The diagnosis of Alport syndrome was based on a combination of characteristic clinical findings (hematuria, lenticonus, retinopathy, etc.), diffuse GBM lamellation in pathological findings, and genetic confirmation of associated mutations (Savige et al., 2013; Kashtan, 2021). Variants of *COL4A5* (X-linked dominant inheritance pattern), including deletion, duplication, substitution, and splicing mutation, account for about 80% of patients with Alport syndrome (Martin et al., 1998). Autosomal recessive and dominant inheritance with *COL4A3* or *COL4A4* mutations accounts for a lesser portion (Martin et al., 1998). Alport patients with *COL4A5* variants are more likely to suffer from proteinuria, focal segmental glomerulosclerosis, and kidney failure than the *COL4A3* and *COL4A4* variants, especially proteinuria which develops at a young age in male patients (Savige and Harraka, 2021). Large deletions, rearrangements, and nonsense mutations of *COL4A5* were reported to be associated with early-onset renal and ocular manifestations (Savige and ColvilleOpinion, 2009). In previous studies, contiguous gene deletions in *COL4A5* and *COL4A6* were reported to be responsible for Alport syndrome–diffuse leiomyomatosis (AS-DL) (Mothes et al., 2002; Murata et al., 2016; Zhou et al., 2021). However, *COL4A6* gene mutation alone was not contributable to Alport syndrome or diffuse leiomyomatosis (Murata et al., 2016). The upregulation of IRS4, a neighboring gene of COL4A5, was considered to be important in the development of leiomyomatosis in patients with AS-DL (Thielen et al., 2003; Mehine et al., 2013). In our case, we consider that the contiguous gene deletions in *COL4A6* and *COL4A5* are the disease-associated mutations that contribute to severe cataract, nephropathy, and possibly uveitis. Although symptoms of leiomyomatosis were not found at this young age, close follow-ups are necessary. Female carriers of these combined deletions reported only a mild clinical presentation, even free of nephropathy (Zhou et al., 2021). This is concordant with our study that although the little boy was affected with ocular and nephropathy, his mother is without any ocular or renal involvement.

Anterior ocular findings associated with Alport syndrome include cataracts, anterior lenticonus, and posterior polymorphous corneal dystrophy (Cheong et al., 1994). Retinal findings such as perimacular dot-and-fleck retinopathy, peripheral confluent retinopathy, retinal thinning, bull’s eye maculopathy, and vitelliform macular detachment have been reported (Fawzi et al., 2009; Savige and ColvilleOpinion, 2009). Uveitis had never been previously reported in Alport syndrome; as a result, we could not differentiate it from infection until the negative results from the metagenomics and microbiological culture were obtained. Tubulointerstitial nephritis and uveitis (TINU) is usually manifested as bilateral sudden onset non-granulomatous anterior uveitis and acute nephritis and can be differentiated from Alport syndrome by typical pathologic findings.

The relationship between Alport syndrome and inflammation has raised interest in recent years. Evidence shows that in chronic kidney disease, the resident kidney cells induce sterile inflammation by activating and producing proinflammatory cytokines and chemokines (Kurts et al., 2013). In our case, the sample from the aqueous humor showed an elevated level of VCAM, IL-6, and IL-8, which indicate a strong inflammatory reaction in the affected eye. We hypothesize that ocular inflammation may result from the breakdown of the blood–retina/aqueous humor barrier, with an autoimmune reaction to abnormal collagen products in the eye.

In an animal study, treatment with the epithelial growth factor receptor (EGFR) inhibitor erlotinib suppressed the expression of inflammatory cytokines in the kidney tissue of AS mice but did not improve the renal pathology (Omachi et al., 2017). In our case, the use of steroids resolved the inflammation in the eye but did not improve the patient’s renal function.

Early diagnosis of Alport syndrome in patients without a related family history is challenging. Extrarenal manifestations such as perceptive hearing loss, lenticonus, and temporal retinal thinning are of limited diagnostic value for these patients as they are generally asymptomatic until a certain age. In our case, the presence of cataract was the original finding. Further diagnosis revealed uveitis. Microscopic hematuria was the only extraocular manifestation and was discovered coincidentally in routine examinations. The confirmative diagnosis was made based on the electron microscopic findings and genetic analysis. The atypical findings (cataracts and uveitis) and asymptomatic findings (hematuria) in patients with Alport syndrome may be underestimated by physicians. We suggest that for patients with ocular manifestations and microscopic hematuria, Alport syndrome should be included in the list of differential diagnoses.

In conclusion, this is the first reported case of non-infectious uveitis associated with Alport syndrome. Anti-inflammatory treatment with steroids is useful for uveitis but not for nephropathy.

## Data Availability

The sequence data reported in this paper have been deposited in the Genome Sequence Archive (GSA) database with the accession no. GVM000399.
